# Association between depression and infertility based on the PHQ-9 score: Analyses of NHANES 2013–2018

**DOI:** 10.1371/journal.pone.0305176

**Published:** 2024-07-22

**Authors:** Li Wan, Sijie He

**Affiliations:** Department of Pharmacy, Maternal and Child Health Hospital of Hubei Province, Tongji Medical College, Huazhong University of Science and Technology, Wuhan, China; Delta State University, NIGERIA

## Abstract

**Background:**

Over the past decade, nationally representative research elucidating the association between depression and infertility has been notably lacking. Our study aimed to investigate the association between depression and infertility among women of childbearing age.

**Methods:**

Our study encompassed 3,654 women aged 18 to 45 years from the National Health and Nutrition Examination Survey (NHANES) 2013–2018. Infertility was defined as a positive response to the query: “Have you attempted to conceive for a minimum of one year without achieving pregnancy?” Depression was evaluated by the Patient Health Questionnaire (PHQ-9) score (range, 0–27). Multiple logistic regression analyses and subgroup analyses stratified by age and race/ethnicity were conducted to investigate the association between depression and infertility. Furthermore, fitted smoothing curves and threshold effect analysis were utilized to depict the nonlinear relationship.

**Results:**

Total PHQ-9 score was associated with infertility in the fully adjusted model (OR 1.04, 95% CI 1.01–1.07, *P* = 0.010), and this relationship exhibited a non-linear pattern, reaching a saturation point at 13, as substantiated by the fitting of smoothed curves. Additionally, the association remained robust when stratified by age but not by race/ethnicity.

**Limitations:**

Cross-sectional design and recall biases.

**Conclusions:**

In this cross-sectional study, depression was associated with infertility among women of childbearing age in the fully adjusted models. This observed association holds potential relevance for clinicians tasked with enhancing psychological well-being during infertility management strategies.

## 1 Introduction

Infertility is the condition characterized by the inability to achieve pregnancy despite engaging in 12 months of consistent unprotected sexual intercourse [1]. The incidence of infertility among women of reproductive age has been approximated to affect one out of every seven couples in Western nations, while in developing countries like China, this condition affects roughly one out of every four couples [2, 3]. Based on the 2017 Global Burden of Disease study, the age-standardized prevalence of infertility in females showed a yearly increment of 0.370% worldwide from 1990 to 2017 [4]. Infertility is highlighted by the Centers for Disease Control and Prevention in the United States (US) as a matter extending beyond the quality of life, bearing significant public health implications encompassing psychological suffering, social stigmatization, financial burden, and marital strife [4]. Globally, infertility imposes a substantial burden on individuals and societies [5]. Moreover, infertility is linked to an elevated risk of developing chronic health conditions like cardiovascular disease [6]. Female infertility has a multifaceted etiology that includes genetic mutations, chromosome abnormalities, lifestyle factors, ovulatory disorders, tubal factors, endometriosis, and unexplained cases [7]. Notably, recent findings demonstrate a positive association between type D personality and infertility, particularly among younger women [8]. Furthermore, contemporary research highlights the efficacy of psychological therapies in alleviating psychological distress and significantly increasing pregnancy rates [9].

Depression constitutes a mood disorder marked by enduring sentiments of sadness and/or anhedonia, alongside concomitant impairments in daily functioning [10]. An escalating prevalence of severe depression emerged among American adults, predominantly discernible within the elderly population (≥ 65 years). Moreover, the incidence of moderate depression exhibited an upswing within the age range of 20 to 39 years [11]. Depression markedly influences the occurrence, financial implications, and consequences of prevalent general medical comorbidities, including diabetes [12]. Moreover, it stands as the foremost risk factor for suicide [13]. Furthermore, associations between depression and oocyte and sperm numbers have sparked increasing concern. A study on 80 Turkish couples assumed a noteworthy link between depression and women’s oocyte retrieval outcomes, as well as oocyte count, revealing that diminished oocyte numbers correlated with heightened depression [14]. Additional investigations have indicated that maternal psychiatric disorders are linked to reduced reproductive success, encompassing diminished oocyte retrieval rates, decreased ongoing pregnancy rates, and perturbation in the stress regulatory system [15]. Moreover, investigators have documented a potential link between self-reported antidepressant usage and an elevated risk of ovulatory infertility [16]. However, despite ovulatory dysfunction and tubal disease are both the prevailing causes of infertility [1], it is inconclusive up to now on the association between infertility and depression. A consensus development assembly, comprising 41 participants representing 11 nations, underscored the significance of delving into research areas that have frequently been disregarded [17]. These encompass the exploration of the emotional and psychological impact on infertility, alongside endeavors to enhance the accessibility of fertility treatment services [17].

Employing the 2013–2018 National Health and Nutritional Examination Surveys (NHANES) dataset, this study aimed to scrutinize the connections between depression and infertility among women aged 18–45 in the US. Additionally, the study sought to ascertain potential age and race/ethnicity-related variations in this relationship.

## 2 Methods

### 2.1 Data source and study population

**Fig 1:**
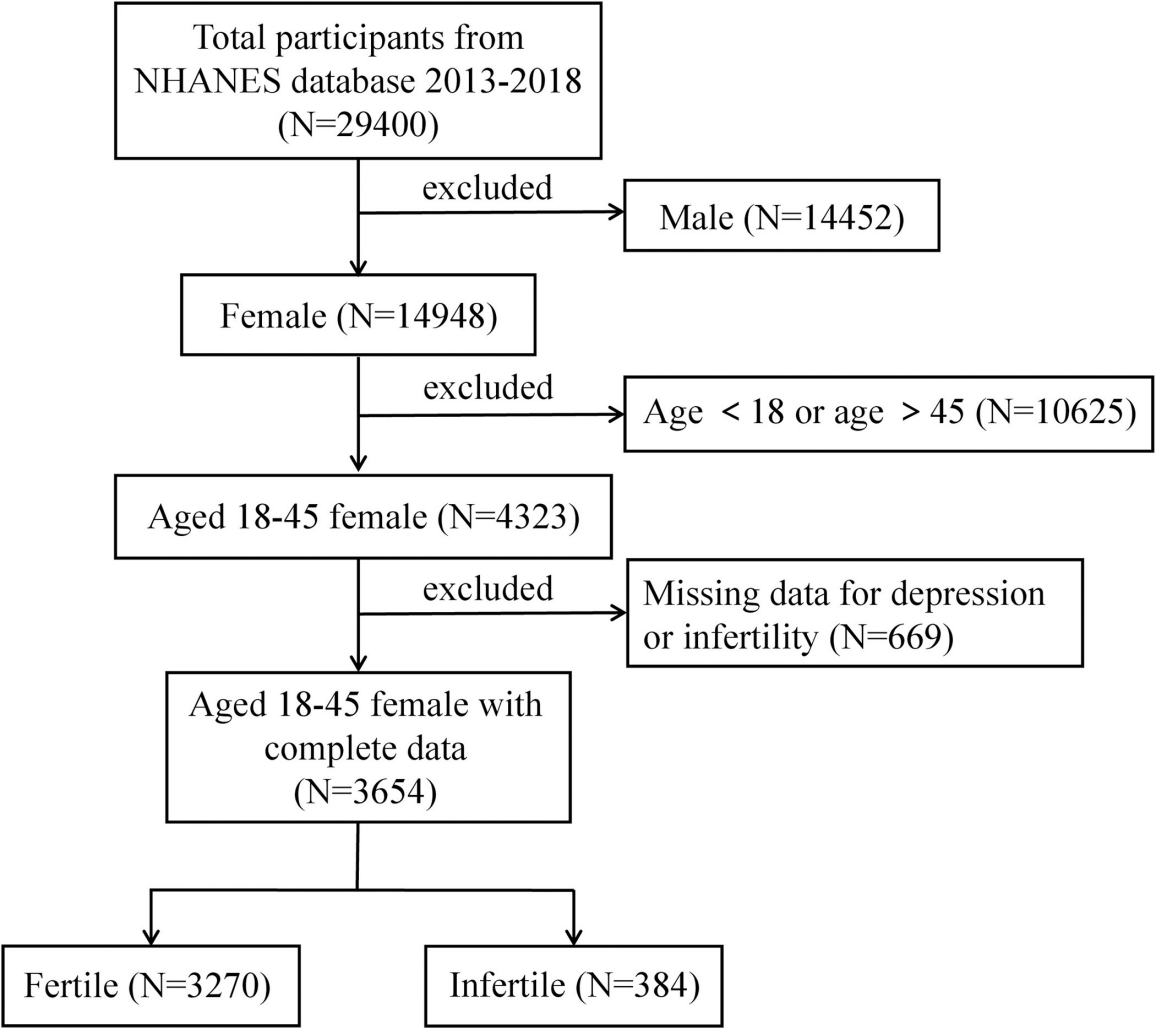
Flow chart of the sample selection from NHANES 2013–2018.

In this cross-sectional study, we utilized publicly accessible data sourced from the NHANES 2013–2018, administered by the National Center for Health Statistics, a division of the Centers for Disease Control and Prevention. NHANES is a representative survey capturing the demographic breadth of the US population. Employing an intricate, multistage, and probabilistic sampling methodology, it furnishes extensive information about the nutritional and health profiles of the general US population [18]. All NHANES data are accessible to the public at www.cdc.gov/nchs/nhanes/. Given the infertility data cycles spanning 2013–2014, 2015–2016, and 2017–2018, the study population comprised participants drawn from the three cycles between 2013–2018. Initially, a total of 29,400 subjects were encompassed within the study. We further proceeded to exclude male participants, individuals aged below 18 years and above 45 years, as well as those who lacked essential infertility information or depression score assessments. Consequently, a total of 3,654 individuals were incorporated. The details of the inclusion-exclusion process are visually depicted in Fig 1. The NHANES study protocol was approved by the National Center for Health Statistics’ Research Ethics Review Board (Protocols 98–12), and all participants provided their written informed consent.

### 2.2 Assessment of infertility

The outcome was the self-reported infertility, obtained from the reproductive health questionnaire. Trained interviewers at the mobile examination center utilized the computer-assisted personal interview system interview to pose the question, “Have you attempted to conceive for a minimum of one year without achieving pregnancy?” Internal consistency checks significantly reduced data entry errors. Individuals answering affirmatively were categorized as individuals with self-reported infertility.

### 2.3 Assessment of depression

The Patient Health Questionnaire (PHQ-9), a standardized depression questionnaire, was used to measure depression [19–21]. It is a nine-item screening tool that inquires about the frequency of depressive symptoms during the past two weeks. The inquiries are derived from the 9 signs and symptoms of depression as delineated in the Diagnostic and Statistical Manual of Mental Disorders, 4th edition. Each of the nine items receives a score between 0 (not at all) and 3 (almost every day), and the sum of the nine item scores ranges from 0 to 27. Scores of 0–4, 5–9, 10–14, and 15–27 denote no, mild, moderate, and severe depression, respectively [22]. To further support our conclusion, the overall score will also be analyzed as a continuous variable.

### 2.4 Assessment of covariates

Age, race/ethnicity, education level, marital status, body mass index (BMI), poverty-income ratio (PIR), diabetes status, smoked at least 100 cigarettes in life, physical activity, cardiovascular disease (CVD), pelvic inflammatory disease (PID), stress urinary incontinence (SUI), substance abuse, and heavy alcohol consumption were selected as covariates based on previous researches [23, 24]. BMI was computed using the formula: weight in kilograms divided by the square of height in meters. PIR, a measure of socioeconomic status, was calculated as the family’s monthly income divided by the Department of Health and Human Services’ poverty thresholds. Smoking status was categorized as “no” or “yes” according to their answer to the question “Have you smoked at least 100 cigarettes in your entire life?” Participants were categorized into two distinct groups based on their physical activity levels: no vigorous activity and vigorous physical activity. CVD was characterized as a combination of 5 self-reported CVD outcomes, encompassing coronary heart disease, congestive heart failure, heart attack, angina pectoris, and stroke. PID was defined as those who answered “yes” to the question “Have you ever been treated for an infection in your fallopian tubes, uterus or ovaries, also called a pelvic infection, pelvic inflammatory disease, or PID?” SUI was determined by self-reporting: “During the past 12 months, have you leaked or lost control of even a small amount of urine with activity like coughing, lifting, or exercise?” Substance abuse was defined by self-reporting: “Have you ever, even once, used marijuana or hashish?” and “Have you ever used cocaine, crack cocaine, heroin, or methamphetamine?” Heavy alcohol consumption was defined as a lifetime history of drinking ≥ 4 to 5 drinks almost every day. For further elaboration on the measurement procedures, additional details can be accessed on the NHANES website (www.cdc.gov/nchs/nhanes/).

### 2.5 Statistical analysis

All analyses accounted for the complex survey design and employed weighting to generate nationally representative estimates. In order to examine baseline characteristics, participants were categorized into two groups based on their infertility status. Means ± standard deviations (SD) were used for describing continuous variables and *p-*values were calculated by the weighted linear regression. Categorical variables were evaluated using the χ^2^ test. The association between infertility prevalence and depression was estimated using multiple logistic regression, and odds ratios (ORs) and 95% confidence intervals (CIs) were presented as a result. We performed tests for trend (“*p*-value for trend”) by entering the median value of each category of depression as a continuous variable in the models [22]. Furthermore, stratification analyses were carried out to assess potential modifications of this association by age and race/ethnicity and their interactions were tested. To quantitatively refine the existing correlations between depression and infertility, allowing us to focus our results, a threshold effects analysis model was applied.

Three models (model 1, model 2, model 3) were constructed using the continuous variable of total PHQ-9 scores as the explanatory model. The individuals were further divided into four groups based on their total PHQ-9 scores, with the no depression group acting as the reference. Another three models (model 4, model 5, model 6) using the categorical form of total PHQ-9 scores were utilized: Model 1 and model 4 acted as the crude models with no covariate adjustments, model 2 and model 5 adjusted for age and race/ethnicity, and model 3 and model 6 additionally adjusted for education level (≤ high school and > high school), marital status (married/living with partner, widowed/divorced/separated and never married), BMI (kg/m^2^, continuous), PIR, diabetes (yes/no), smoked at least 100 cigarettes in life (yes/no), physical activity (vigorous activity, yes/no), CVD, PID, SUI, substance abuse, and heavy alcohol consumption.

Statistical analyses were performed using R (version 3.4.3, www.r-project.org, AT&T BellLaboratories, New Zealand), Stata (version 16.0, www.stata.com, StataCorp, America), and Empower Stats (version 4.2, www.empowerstats.com, X&Y solutions, inc. Boston, Massachusetts). A two-sided *P* value of < 0.05 was considered statistically significant.

## 3 Results

### 3.1 Baseline characteristics of participants

Table 1 presents the weighted baseline characteristics of the study’s participants, which consisted of 3,654 females aged 18 to 45. Within this assemblage, 384 individuals were identified as suffering from infertility, whereas 3,270 were not, with an average age of 31.453 years. Infertility prevalence was notably higher among women who were older at the time of the survey (35.37 years vs. 30.94 years, *P* < 0.001), had a higher BMI (32.20 kg/m^2^ vs 28.90 kg/m^2^, *P* < 0.001), and had a greater family income (PIR: 2.81 vs 2.61, *P* = 0.028). Additionally, those suffering from infertility tended to be never married (77.15% vs. 57.62%, *P* < 0.001), coupled with an elevated likelihood of diabetes (8.13% vs 2.96%, *P* < 0.001), PID (8.97% vs 4.00%, *P* < 0.001), SUI (45.10% vs 31.34%, *P* < 0.001), and heavy alcohol consumption (12.30% vs 7.14%, *P* < 0.001) and a history of smoking at least 100 cigarettes during their lifetime (38.31% vs 29.63%, *P* < 0.001). In addition, the unweighted frequency and the percentage distribution were displayed in the S1 Table.

**Table 1:** Weighted characteristics of the study population in the NHANES 2013–2018.

Characteristic	Total (n = 3654)	No infertility (n = 3270)	Infertility (n = 384)	*P* value
**Age (years)**	31.45 ± 8.12	30.94 ± 8.11	35.37 ± 7.10	< 0.001
**Race/Ethnicity (%)**				0.067
Mexican American	12.01	12.23	10.28	
Non-Hispanic white	56.09	55.30	62.06	
Non-Hispanic black	13.43	13.59	12.22	
Other races	18.48	18.88	15.44	
**Education level (%)**				0.740
≤ High school	30.72	30.82	30.02	
> high school	69.28	69.18	69.98	
**Marital status (%)**				< 0.001
Married/Living with partner	29.64	32.14	11.83	
Widowed/Divorced/Separated	10.33	10.24	11.02	
Never Married	60.03	57.62	77.15	
**Diabetes (%)**				< 0.001
No	96.45	97.04	91.87	
Yes	3.5535	2.96	8.13	
**Physical activity (%)**				0.687
No vigorous activity	82.27	82.18	82.98	
Vigorous physical activity	17.73	17.82	17.02	
**Smoked at least 100 cigarettes in life (%)**				< 0.001
No	69.37	70.37	61.69	
Yes	30.63	29.63	38.31	
**CVD (%)**				0.061
No	98.18	98.34	97.03	
Yes	1.83	1.66	2.98	
**PID (%)**				< 0.001
No	95.42	96.00	91.03	
Yes	4.58	4.00	8.97	
**SUI (%)**				< 0.001
No	67.05	68.66	54.90	
Yes	32.95	31.34	45.10	
**Substance abuse (%)**				0.608
No	44.68	44.84	43.51	
Yes	55.32	55.16	56.49	
**Heavy alcohol consumption (%)**				< 0.001
No	92.23	92.86	87.70	
Yes	7.77	7.14	12.30	
**Depression (%)**				< 0.001
No depression	72.75	73.72	65.39	
Mild depression	16.81	16.42	19.79	
Moderate depression	7.05	6.79	9.01	
Severe depression	3.39	3.07	5.81	
**PIR**	2.64 ± 1.65	2.61 ± 1.66	2.81 ± 1.63	0.028
**BMI (kg/m** ^**2**^ **)**	29.28 ± 8.31	28.90 ± 8.07	32.20 ± 9.46	< 0.001
**Total PHQ-9 score**	3.57 ± 4.29	3.46 ± 4.19	4.44 ± 4.86	< 0.001

Mean ± SD for continuous variables: *P* value was calculated by the weighted linear regression model; % for categorical variables: *P* value was calculated by weighted χ^2^ test. NHANES, National Health and Nutrition Examination Survey; CVD, cardiovascular disease; PID, pelvic inflammatory disease; SUI, stress urinary incontinence; PIR, poverty-income ratio; BMI, body mass index.

### 3.2 Association between depression and infertility

We employed three models to examine the association between depression and infertility. Table 2 presents the corresponding ORs with their 95% CIs and associated *P*-values. The positive association remained significant after adjusting for all of the covariates, when the total PHQ-9 score was treated as a continuous variable (OR 1.04, 95% CI 1.01–1.07, *P* = 0.010). Moreover, compared to the no depression group, the moderate depression group (OR 2.05, 95% CI 1.30–3.22, *P* = 0.002) exhibited an elevated prevalence of infertility in the fully adjusted model. The mild depression group was related to an elevated infertility prevalence in model 4 (OR 1.36, 95% CI 1.04–1.79, *P* = 0.024) and model 5 (OR 1.40, 95% CI 1.07–1.84, *P* = 0.015), but not in model 6. Severe depression was positively associated with a higher infertility prevalence only in model 4 (OR 1.76, 95% CI 1.07–2.88, *P* = 0.026), but not in model 5 and or model 6.

**Table 2:** Association between depression and infertility in NHANES 2013–2018.

Continuous	Model 1		Model 2		Model 3	
	OR (95%CI)	*P* value	OR (95%CI)	*P* value	OR (95%CI)	*P* value
**Total PHQ-9 score**	1.05 (1.02, 1.07)	< 0.001	1.05 (1.02, 1.07)	< 0.001	1.04 (1.01, 1.07)	0.010
Categorical groups	**Model 4**		**Model 5**		**Model 6**	
	**OR (95%CI)**	***P* value**	**OR (95%CI)**	***P* value**	**OR (95%CI)**	***P* value**
No depression	reference		reference		reference	
Mild depression	1.36 (1.04, 1.79)	0.024	1.40 (1.07, 1.84)	0.015	1.28 (0.93, 1.78)	0.135
Moderate depression	1.76 (1.21, 2.58)	0.003	1.74 (1.18, 2.56)	0.005	2.05 (1.30, 3.22)	0.002
Severe depression	1.76 (1.07, 2.88)	0.026	1.65 (1.00, 2.73)	0.051	1.32 (0.71, 2.43)	0.378
*P* for trend	< 0.001		< 0.001		0.013	

Model 1 and model 4 adjusted for none. Model 2 and model 5 adjusted for age and race/ethnicity. Model 3 and model 6 adjusted for age, race/ethnicity, education level, marital status, BMI, PIR, diabetes, smoked at least 100 cigarettes in life, physical activity, CVD, PID, SUI, substance abuse, and heavy alcohol consumption. NHANES, National Health and Nutrition Examination Survey; PHQ-9, Patient Health Questionnaire 9; OR, odds ratio; CI, confidence interval.

### 3.3 Subgroup analysis

The interaction test demonstrated that there was a significant interaction between depression and race/ethnicity (*P* for interaction = 0.038), but no significant interaction between depression and age (*P* for interaction = 0.524) (Table 3), suggesting that infertility prevalence associated with depression changes over race/ethnicity but not over age (Table 3). Following stratification by race/ethnicity, this statistically significant association was only evident in Mexican Americans (OR = 1.10, 95% CI: 1.02–1.19, *P* = 0.017) and other races (OR = 1.07, 95% CI: 1.01–1.13, *P* = 0.020) (Table 3).

**Table 3:** Association between depression and infertility stratified by age or race/ethnicity in NHANES 2013–2018.

	OR (95%CI)	*P* value	*P* for interaction
**Age**			0.524
18–24 years	1.05 (0.96, 1.15)	0.253	
24–31 years	1.09 (1.03, 1.17)	0.007	
31–39 years	1.04 (0.99, 1.10)	0.086	
39–45 years	1.02 (0.97, 1.08)	0.372	
**Race/Ethnicity (%)**			0.038
Mexican American	1.10 (1.02, 1.19)	0.017	
Non-Hispanic White	0.93 (0.81, 1.07)	0.340	
Non-Hispanic Black	1.04 (1.00, 1.09)	0.084	
Other races	1.07 (1.01, 1.13)	0.020	

Age or race/ethnicity, education level, marital status, BMI, PIR, diabetes, smoked at least 100 cigarettes in life, physical activity, CVD, PID, SUI, substance abuse, and heavy alcohol consumption were adjusted. NHANES, National Health and Nutrition Examination Survey; OR, odds ratio; CI, confidence interval.

Upon categorizing of total PHQ-9 score into distinct depression levels, the ORs (95% CI) resulting from age-stratified and race/ethnicity-stratified analyses were presented in S2 and S3 Tables, respectively. After adjusting for potential confounders, the infertility prevalence of participants with moderate depression significantly surpassed that of non-depressed counterparts in both the 24–31 years (*P* <0.001) and 31–39 years age groups (*P* = 0.009) (S2 Table). Particularly noteworthy, participants experiencing severe depression exhibited a 5.488-fold greater infertility prevalence compared to their non-depressed counterparts in the 18–24 years age group (S2 Table). Moreover, individuals with moderate depression exhibited heightened infertility prevalence relative to those without depression only in non-Hispanic black group (*P* = 0.002) group (S3 Table). Participants exhibiting severe depression demonstrated an elevated infertility risk in comparison to their non-depressed counterparts only within the Mexican American population (*P* = 0.036) (S3 Table).

**Fig 2:**
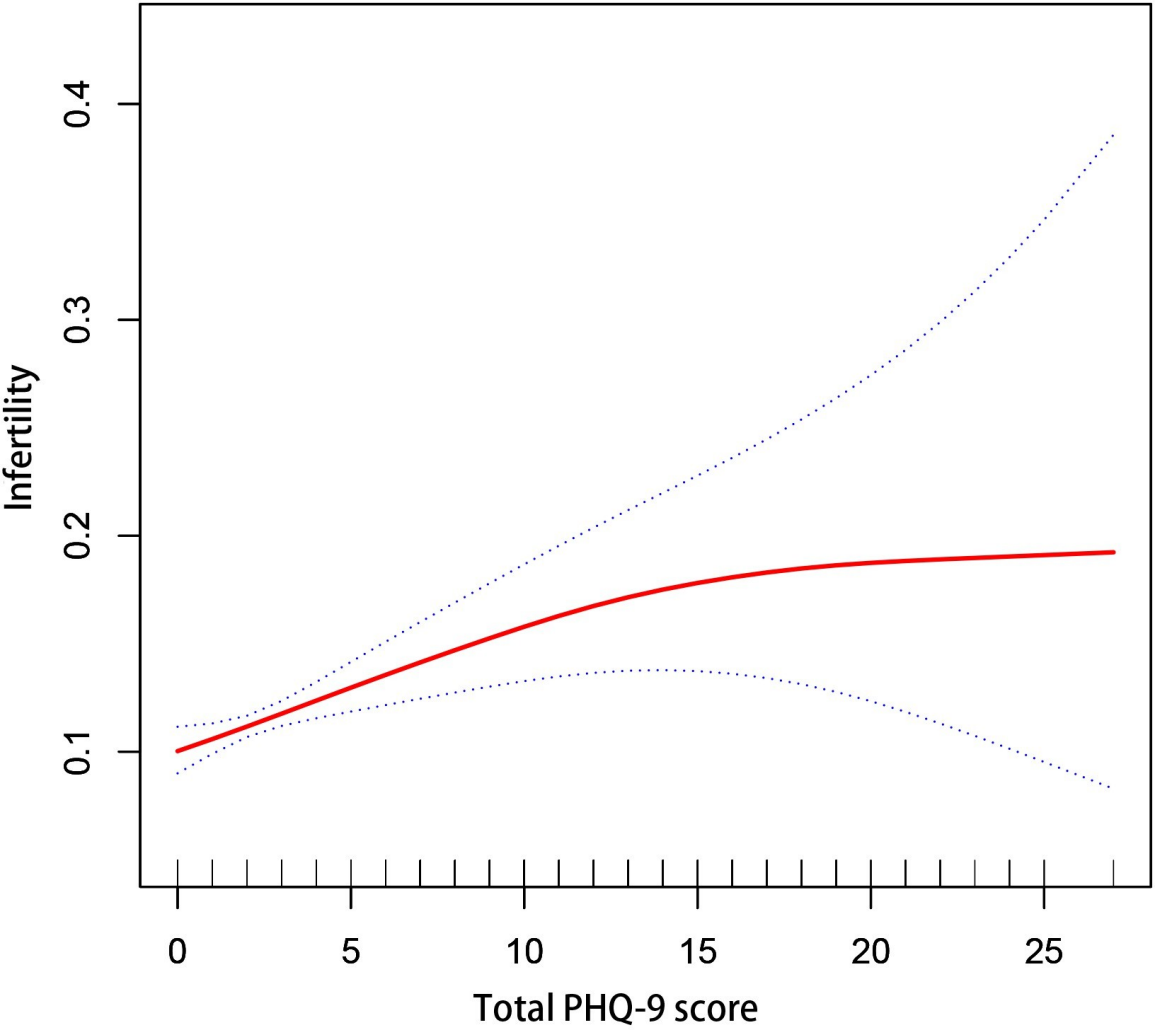
The association between depression and infertility. The solid line represents the smooth curve fit between variables. Dotted line represents the 95% of confidence interval from the fit. Age, race/ethnicity, education level, marital status, BMI, PIR, diabetes, smoked at least 100 cigarettes in life, physical activity, CVD, PID, SUI, substance abuse, and heavy alcohol consumption were adjusted.

We further observed a saturation effect between the total PHQ-9 score and infertility when conducting smoothing curve fitting in the fully adjusted model (Fig 2). A threshold effect analysis model was employed, revealing the turning point at 13 (Table 4), implying that there was still a correlation between the total PHQ-9 score and infertility before but not after the inflection point (Table 4).

**Table 4:** Saturation effect analysis of depression on infertility of all participants.

Infertility	Model: saturation effect analysis
Turning point (K)	13
< K, effect 1	1.07 (1.03, 1.11) <0.001
> K, effect 2	0.94 (0.84, 1.05) 0.283
Log likelihood ratio	0.045

Age, race/ethnicity, education level, marital status, BMI, PIR, diabetes, smoked at least 100 cigarettes in life, physical activity, CVD, PID, SUI, substance abuse, and heavy alcohol consumption were adjusted.

## 4 Discussion

In this study, our findings revealed that heightened total PHQ-9 score exhibited a robust positive association with escalated infertility susceptibility. Besides, moderate depression participants presented higher infertility prevalence compared to people without depression. Subsequent subgroup analysis demonstrated that in the fully adjusted model, these relationships between total PHQ-9 score and infertility were robust in the age subgroup, but pronounced among Mexican American and other races participants. Remarkably, saturation analysis contributed to the discovery of the turning point at 13.

According to numerous researches, depressive disorders are extremely prevalent among women with infertility [8, 25–28]. Prior to their initial infertility clinic visit, 122 women were examined using a systematic psychiatric assessment. The results were startling: 17% of the women had been diagnosed with depression [25]. These results have been supported by further studies. Major depression was discovered to be the most prevalent mood disorder among 545 couples that visited a reproductive clinic in Sweden [26]. An earlier cross-sectional study involving 324 women aged 18–44 years revealed that Type D personality and depression were considerably more common in the infertile group than the fertile group [8]. Another clinical and online based cross-sectional survey with a sample size of 225 from Hungary also indicated that infertile women were more likely to experience depression and anxiety-related symptoms than fertile women [29]. Nevertheless, only a few studies have assessed the US population.

As far as we know, only two cross-sectional researches have been conducted in the US pertaining to the association between depression and infertility. One study, involving a cohort of 2,920 women aged 18 to 45 years, revealed a link between a history of depressive symptoms and an increased susceptibility to female infertility [30]. Using data from the National Survey of Fertility Barriers in the US, an additional study investigated the relationships between meeting medical criteria for infertility and self-perceived fertility issues with depressive symptoms. The classification of meeting medical criteria for infertility encompassed individuals attempting conception yet not achieving pregnancy within 12 months. Consequently, both infertility criteria exhibited statistically significant associations with depressive symptoms, even after adjustment for pertinent covariates, aligning harmoniously with our study’s outcomes. Nonetheless, it is noteworthy that these investigations relied on datasets from earlier periods, specifically the years 1981–1990 and 2004–2007, respectively. Considering the evolving social context surrounding infertility and depression, potential alterations in the interplay between these phenomena warrant consideration [31]. Our study focused on the US demographic during the interval of 2013–2018, which represents the latest available infertility data within the NHANES database. This temporal scope holds the potential to enhance and complement prior work. Furthermore, previous investigations omitted exploration into the connection between depression and infertility across distinct subgroups. Through the subgroup analysis, we unveiled disparities in these associations based on age and race/ethnicity categories. Several factors have been demonstrated to be intertwined with depression, responses to infertility, or both [31]. Previous research has substantiated the influence of age and race/ethnicity on infertility prevalence [32–35]. Depression also exhibits conspicuous disparities across socioeconomic, racial, and gender strata [36, 37]. Drawing upon data derived from the Humana and Optum databases, a cross-sectional study unveiled disparities in the prevalence of treatment-resistant depression, stratified by sex, race, and age. Notably, heightened prevalence was observed among females, white individuals, and those aged 45–64 years [38]. Nevertheless, scant exploration has delved into variances rooted in age and race/ethnicity concerning the interplay between depression and infertility. Given this, it is prudent to approach these findings, while underscoring the need for meticulously designed prospective studies in this realm.

Hypotheses had been posited suggesting that psychiatric symptoms may serve as both causal factors for infertility or consequences thereof, or potentially encompassing both scenarios [39]. Discernible inference had been drawn that infertility contributes to the manifestation of psychological stress [9]. Nevertheless, the precise mechanisms underlying depression’s impact on infertility remain inadequately explored, with no definitive conclusions as of yet. Prior studies have proposed direct mechanisms linking depression to infertility, encompassing psychoendocrinological pathways characterized by heightened levels of prolactin and cortisol, psychoimmunological pathways involving compromised immune defenses, and behavioral pathways encompassing diminished libido and an increased propensity for smoking [30, 40]. Derived from a cross-sectional study, the findings revealed an association between depressive disorder in young individuals and a proclivity for engaging in precarious sexual behaviors, including multiple partners and inconsistent or nonexistent condom usage. These behaviors correspondingly heightened the susceptibility to contracting sexually transmitted diseases, which could potentially contribute to subsequent infertility [41].

The strength of this study resides in its employment of extensive nationally representative datasets, accompanied by stratified analyses based on age and race/ethnicity categories and a comprehensive assessment of the association between depression and infertility through saturation analysis. However, this study presents several limitations. Initially, the cross-sectional nature of the NHANES dataset restricts our capacity to establish causal links or furnish extended longitudinal data concerning the included individuals. Secondly, infertility diagnosis relied on self-reported data, which carries the potential for recall bias. Thirdly, our analysis encompassed only non-Hispanic white, non-Hispanic black, and Mexican American participants for examinations across distinct race/ethnicity groups due to constraints in sample size for other race/ethnicity groups. Finally, although adjustments were made for certain potential confounding variables, not all residual confounding elements could be fully addressed.

In summary, our findings indicate an association between elevated total PHQ-9 score and heightened infertility prevalence among childbearing women in the US, with this relationship being moderated by race/ethnicity but not by age. Our discoveries bear profound implications for clinical application. Through the evaluation and enhancement of the psychological well-being of patients, particularly those afflicted by depression, there exists the potential to ameliorate infertility metrics. To give more precise and efficient prevention and treatment choices for infertility, it is critically necessary in the future for additional randomized controlled trials or cohort studies to verify this conclusion.

## Supporting information

S1 Table Characteristics of the study population in the NHANES 2013–2018. Mean ± SD for continuous variables: *P* value was calculated by one-way ANOVA; % for categorical variables: *P* value was calculated by χ^2^ test. NHANES, National Health and Nutrition Examination Survey; PIR, poverty-income ratio; BMI, body mass index.
(DOCX)

S2 Table Association between depression and infertility stratified by age in NHANES 2013–2018. Model 1 adjusted for none. Model 2 adjusted for race/ethnicity. Model 3 adjusted for race/ethnicity, education level, marital status, BMI, PIR, diabetes, smoked at least 100 cigarettes in life, physical activity, CVD, PID, SUI, substance abuse, and heavy alcohol consumption. NHANES, National Health and Nutrition Examination Survey; PHQ-9, Patient Health Questionnaire 9; OR, odds ratio; CI, confidence interval.
(DOCX)

S3 Table Association between depression and infertility stratified by race/ethnicity in NHANES 2013–2018. Model 1 adjusted for none. Model 2 adjusted for age. Model 3 adjusted for age, education level, marital status, BMI, PIR, diabetes, smoked at least 100 cigarettes in life, physical activity, CVD, PID, SUI, substance abuse, and heavy alcohol consumption. NHANES, National Health and Nutrition Examination Survey; PHQ-9, Patient Health Questionnaire 9; OR, odds ratio; CI, confidence interval.
(DOCX)
