# Supplementary material for: Association between depression and infertility based on the PHQ-9 score: Analyses of NHANES 2013–2018
Source: PLoS One. 2024 Jul 22;19(7):e0305176. doi: 10.1371/journal.pone.0305176 (PMC11262654; doi:10.1371/journal.pone.0305176)
Supplement: S1 Table — Mean ± SD for continuous variables: P value was calculated by one-way ANOVA; % for categorical variables: P value was calculated by χ2 test. NHANES, National Health and Nutrition Examination Survey; PIR, poverty-income ratio; BMI, body mass index. (DOCX) [file pone.0305176.s001.docx]

S1 Table Characteristics of the study population in the NHANES 2013-2018.

| **Characteristic** | Total  （n=3654） | No infertility  （n=3270） | Infertility  （n=384） | *P* value |
| --- | --- | --- | --- | --- |
| **Age (years)** | 31.18 ± 8.43 | 30.75 ± 8.46 | 34.83 ± 7.25 | <0.001 |
| **Race/Ethnicity (%)** |  |  |  | 0.081 |
| Mexican American | 645 (17.652%) | 583 (17.829%) | 62 (16.146%) |  |
| Non-Hispanic white | 1187 (32.485%) | 1040 (31.804%) | 147 (38.281%) |  |
| Non-Hispanic black | 806 (22.058%) | 726 (22.202%) | 80 (20.833%) |  |
| Other races | 1016 (27.805%) | 921 (28.165%) | 95 (24.740%) |  |
| **Education level (%)** |  |  |  | <0.001 |
| ≤ High school | 1132 (30.98%) | 1005 (30.73%) | 127 (33.07%) |  |
| > high school | 2108 (57.69%) | 1858 (56.82%) | 250 (65.10%) |  |
| **Marital status (%)** |  |  |  | <0.001 |
| Married/Living with partner | 993 (27.18%) | 937 (28.65%) | 56 (14.58%) |  |
| Widowed/Divorced/Separated | 361 (9.88%) | 315 (9.63%) | 46 (11.98%) |  |
| Never Married | 1886 (51.62%) | 1611 (49.27%) | 275 (71.62%) |  |
| **Diabetes (%)** |  |  |  | <0.001 |
| No | 3447 (94.34%) | 3103 (94.89%) | 344 (89.58%) |  |
| Yes | 153 (4.19%) | 120 (3.67%) | 33 (8.59%) |  |
| **Physical activity (%)** |  |  |  | 0.701 |
| No vigorous activity | 3037 (83.11%) | 2721 (83.21%) | 316 (82.29%) |  |
| Vigorous physical activity | 616 (16.86%) | 548 (16.76%) | 68 (17.71%) |  |
| **Smoked at least 100 cigarettes in life** |  |  |  | <0.001 |
| No | 2679 (73.32%) | 2430 (74.31%) | 249 (64.84%) |  |
| Yes | 973 (26.63%) | 838 (25.63%) | 135 (35.16%) |  |
| **Depression (%)** |  |  |  | 0.002 |
| No depression | 2653 (72.61%) | 2404 (73.52%) | 249 (64.84%) |  |
| Mild depression | 638 (17.46%) | 559 (17.09%) | 79 (20.57%) |  |
| Moderate depression | 233 (6.38%) | 197 (6.02%) | 36 (9.38%) |  |
| Severe depression | 130 (3.56%) | 110 (3.36%) | 20 (5.21%) |  |
| **PIR** | 2.27 ± 1.60 | 2.24 ± 1.59 | 2.51 ± 1.64 | 0.002 |
| **BMI (kg/m^2^)** | 29.37 ± 8.40 | 29.04 ± 8.20 | 32.18 ± 9.54 | <0.001 |
| **Total PHQ-9 score** | 3.54 ± 4.33 | 3.44 ± 4.26 | 4.42 ± 4.80 | <0.001 |

Mean ± SD for continuous variables: *P* value was calculated by one-way ANOVA; % for categorical variables: *P* value was calculated by χ^2^ test.

NHANES, National Health and Nutrition Examination Survey; PIR, poverty-income ratio; BMI, body mass index.
