# Supplementary material for: Association between depression and infertility based on the PHQ-9 score: Analyses of NHANES 2013–2018
Source: PLoS One. 2024 Jul 22;19(7):e0305176. doi: 10.1371/journal.pone.0305176 (PMC11262654; doi:10.1371/journal.pone.0305176)
Supplement: S2 Table — Model 1 adjusted for none. Model 2 adjusted for race/ethnicity. Model 3 adjusted for race/ethnicity, education level, marital status, BMI, PIR, diabetes, smoked at least 100 cigarettes in life, physical activity, CVD, PID, SUI, substance abuse, and heavy alcohol consumption. NHANES, National Health and Nutrition Examination Survey; PHQ-9, Patient Health Questionnaire 9; OR, odds ratio; CI, confidence interval. (DOCX) [file pone.0305176.s002.docx]

S2 Table Association between depression and infertility stratified by age in NHANES 2013-2018.

| **Infertility** | **Model 1** | | **Model 2** | | **Model 3** | |
| --- | --- | --- | --- | --- | --- | --- |
|  | **OR (95%CI)** | ***P* value** | **OR (95%CI)** | ***P* value** | **OR (95%CI)** | ***P* value** |
| **18-24 years** | | | | | | |
| No depression | Reference | | Reference | | Reference | |
| Mild depression | 1.369 (0.596, 3.146) | 0.460 | 1.322 (0.573, 3.052) | 0.513 | 1.377 (0.482, 3.391) | 0.550 |
| Moderate depression | 0.985 (0.225, 4.308) | 0.984 | 0.943 (0.214, 4.164) | 0.939 | 0.284 (0.033, 2.481) | 0.255 |
| Severe depression | 6.122 (2.290, 16.366) | < 0.001 | 6.503 (2.392, 17.682) | < 0.001 | 6.488 (1.346, 31.271) | 0.020 |
| **24-31 years** | | | | | | |
| No depression | Reference | | Reference | | Reference | |
| Mild depression | 1.651 (0.904, 3.016) | 0.103 | 1.607 (0.876, 2.946) | 0.125 | 1.448 (0.716, 2.929) | 0.303 |
| Moderate depression | 3.612 (1.688, 7.731) | < 0.001 | 3.770 (1.741, 8.162) | < 0.001 | 5.371 (2.138, 13.494) | <0.001 |
| Severe depression | 4.224 (1.322, 13.497) | 0.015 | 4.137 (1.270, 13.475) | 0.018 | 1.404 (0.274, 7.183) | 0.684 |
| **31-39 years** | | | | | | |
| No depression | Reference | | Reference | | Reference | |
| Mild depression | 1.298 (0.790, 2.135) | 0.303 | 1.297 (0.788, 2.134) | 0.306 | 1.515 (0.853, 2.688) | 0.156 |
| Moderate depression | 1.426 (0.719, 2.830) | 0.310 | 1.377 (0.691, 2.744) | 0.364 | 2.709 (1.280, 5.734) | 0.009 |
| Severe depression | 0.757 (0.263, 2.174) | 0.605 | 0.733 (0.254, 2.113) | 0.566 | 0.852 (0.253, 2.868) | 0.796 |
| **39-45 years** | | | | | | |
| No depression | Reference | | Reference | | Reference | |
| Mild depression | 1.440 (0.925, 2.240) | 0.106 | 1.508 (0.964, 2.359) | 0.072 | 1.288 (0.693, 2.393) | 0.423 |
| Moderate depression | 1.680 (0.876, 3.223) | 0.119 | 1.708 (0.883, 3.304) | 0.112 | 2.146 (0.829, 5.554) | 0.115 |
| Severe depression | 1.139 (0.464, 2.797) | 0.777 | 1.120 (0.451, 2.782) | 0.807 | 1.240 (0.411, 3.740) | 0.703 |

Model 1 adjusted for none. Model 2 adjusted for race/ethnicity. Model 3 adjusted for race/ethnicity, education level, marital status, BMI, PIR, diabetes, smoked at least 100 cigarettes in life, physical activity, CVD, PID, SUI, substance abuse, and heavy alcohol consumption.

NHANES, National Health and Nutrition Examination Survey; PHQ-9, Patient Health Questionnaire 9; OR, odds ratio; CI, confidence interval.
