# Supplementary material for: Association between depression and infertility based on the PHQ-9 score: Analyses of NHANES 2013–2018
Source: PLoS One. 2024 Jul 22;19(7):e0305176. doi: 10.1371/journal.pone.0305176 (PMC11262654; doi:10.1371/journal.pone.0305176)
Supplement: S3 Table — Model 1 adjusted for none. Model 2 adjusted for age. Model 3 adjusted for age, education level, marital status, BMI, PIR, diabetes, smoked at least 100 cigarettes in life, physical activity, CVD, PID, SUI, substance abuse, and heavy alcohol consumption. NHANES, National Health and Nutrition Examination Survey; PHQ-9, Patient Health Questionnaire 9; OR, odds ratio; CI, confidence interval. (DOCX) [file pone.0305176.s003.docx]

S3 Table Association between depression and infertility stratified by race/ethnicity in NHANES 2013-2018.

| **Infertility** | **Model 1** | | **Model 2** | | **Model 3** | |
| --- | --- | --- | --- | --- | --- | --- |
|  | **OR (95%CI)** | ***P* value** | **OR (95%CI)** | ***P* value** | **OR (95%CI)** | ***P* value** |
| **Mexican American** | | | | | | |
| No depression | Reference | | Reference | | Reference | |
| Mild depression | 1.156 (0.588, 2.272) | 0.674 | 1.136 (0.577, 2.235) | 0.713 | 1.395 (0.619, 3.143) | 0.422 |
| Moderate depression | 1.586 (0.528, 4.762) | 0.411 | 1.520 (0.504, 4.583) | 0.457 | 1.237 (0.256, 5.975) | 0.791 |
| Severe depression | 2.426 (0.780, 7.542) | 0.126 | 2.492 (0.797, 7.795) | 0.116 | 6.082 (1.122, 32.972) | 0.036 |
| **Non-Hispanic white** | | | | | | |
| No depression | Reference | | Reference | | Reference | |
| Mild depression | 1.144 (0.725, 1.805) | 0.564 | 1.226 (0.768, 1.956) | 0.393 | 1.214 (0.710, 2.078) | 0.479 |
| Moderate depression | 1.272 (0.695, 2.327) | 0.435 | 1.431 (0.770, 2.661) | 0.257 | 2.062 (0.993, 4.281) | 0.052 |
| Severe depression | 1.508 (0.686, 3.316) | 0.307 | 1.306 (0.584, 2.920) | 0.516 | 1.563 (0.611, 3.997) | 0.351 |
| **Non-Hispanic black** | | | | | | |
| No depression | Reference | | Reference | | Reference | |
| Mild depression | 2.309 (1.323, 4.030) | 0.003 | 2.387 (1.362, 4.184) | 0.002 | 1.919 (0.962, 3.828) | 0.064 |
| Moderate depression | 4.120 (2.085, 8.141) | < 0.001 | 3.856 (1.941, 7.660) | < 0.001 | 3.712 (1.615, 8.535) | 0.002 |
| Severe depression | 2.943 (1.061, 8.165) | 0.038 | 2.864 (1.025, 8.002) | 0.045 | 1.316 (0.325, 5.330) | 0.701 |
| **Other races** | | | | | | |
| No depression | Reference | | Reference | | Reference | |
| Mild depression | 1.248 (0.722, 2.159) | 0.427 | 1.273 (0.728, 2.227) | 0.397 | 1.152 (0.532, 2.496) | 0.719 |
| Moderate depression | 0.958 (0.334, 2.750) | 0.936 | 1.002 (0.342, 2.934) | 0.997 | 1.094 (0.321, 3.734) | 0.886 |
| Severe depression | 1.006 (0.299, 3.379) | 0.993 | 0.960 (0.279, 3.304) | 0.948 | 0.284 (0.040, 2.025) | 0.209 |

Model 1 adjusted for none. Model 2 adjusted for age. Model 3 adjusted for age, education level, marital status, BMI, PIR, diabetes, smoked at least 100 cigarettes in life, physical activity, CVD, PID, SUI, substance abuse, and heavy alcohol consumption.

NHANES, National Health and Nutrition Examination Survey; PHQ-9, Patient Health Questionnaire 9; OR, odds ratio; CI, confidence interval.
